# Low serum estradiol levels are related to *Mycobacterium avium* complex lung disease: a cross-sectional study

**DOI:** 10.1186/s12879-019-4668-x

**Published:** 2019-12-16

**Authors:** Yoshifumi Uwamino, Tomoyasu Nishimura, Yasunori Sato, Eiko Tamizu, Takanori Asakura, Shunsuke Uno, Masaaki Mori, Hiroshi Fujiwara, Makoto Ishii, Hiroshi Kawabe, Mitsuru Murata, Naoki Hasegawa

**Affiliations:** 1 Department of Laboratory Medicine, Keio University School of Medicine, 35 Shinanomachi, Shinjuku-ku, Tokyo, Japan; 2 Department of Infectious Diseases, Keio University School of Medicine, 35 Shinanomachi, Shinjuku-ku, Tokyo, Japan; 3 Keio University Health Center, 35 Shinanomachi, Shinjuku-ku, Tokyo, 160-8582 Japan; 4 Department of Preventive Medicine and Public Health, Keio University School of Medicine, 35 Shinoanomachi, Shinjuku-ku, Tokyo, Japan; 5 Division of Pulmonary Medicine, Department of Medicine, Keio University School of Medicine, 35 Shinanomachi, Shinjuku-ku, Tokyo, Japan; 6 Department of Internal Medicine, Keio University School of Medicine, 35 Shinanomachi, Shinjuku-ku, Tokyo, Japan

**Keywords:** Estradiol, *Mycobacterium avium* complex, Postmenopausal women

## Abstract

**Background:**

The risk factors for *Mycobacterium avium* complex lung disease (MAC-LD) are not well known. We hypothesized that low serum estradiol (E2) levels are related to MAC-LD as most patients with MAC-LD are postmenopausal women.

**Methods:**

This cross-sectional study compared patients with MAC-LD and healthy controls. Study subjects were postmenopausal women aged 65 years or younger. Serum testosterone, dehydroepiandrosterone sulfate (DHEA-S), and E2 levels were measured and categorized as high or low based on median levels. We performed multivariate analysis, receiver operating characteristic (ROC) curve analysis, and age- and body mass index (BMI)-matched subgroup analysis to evaluate the association between low serum E2 levels and MAC-LD.

Additionally, using blood samples obtained for other clinical studies, the levels of sex steroid hormones were compared between age- and BMI-matched MAC-LD and bronchiectasis female patients without non-tuberculosis mycobacterial infections (non-NTM BE).

**Results:**

Forty-two patients with MAC-LD and 91 healthy controls were included. The median E2 (2.20 pg/mL vs. 15.0 pg/mL, *p* < 0.001), testosterone (0.230 ng/L vs. 0.250 ng/L, *p* = 0.005), and DHEA-S (82.5 μg/dL vs. 114.0 μg/dL, *p* < 0.001) levels were lower in the MAC-LD group than in the control group. Multivariate analysis revealed that low serum E2 (adjusted odds ratio = 34.62, 95% confidence interval = 6.02–199.14) was independently related to MAC-LD, whereas low DHEA-S and testosterone were not. ROC analysis illustrated a strong relationship between low serum E2 levels and MAC-LD (area under the curve = 0.947, 95% confidence interval = 0.899–0.995). Even the age- and BMI-matched subgroup analysis of 17 MAC-LD patients and 17 healthy controls showed lower serum E2 in MAC-LD patients than in healthy controls. Additionally, serum E2 levels of 20 MAC-LD patients were lower than plasma E2 levels of 11 matched non-NTM BE patients (1.79 pg/mL vs. 11.0 pg/mL, *p* < 0.001).

**Conclusions:**

Low serum E2 levels were strongly related to MAC-LD in postmenopausal women.

## Background

The incidence of pulmonary nontuberculous mycobacterial (NTM) infection continues to increase globally [1–3]. Although *Mycobacterium avium* complex (MAC) lung disease (MAC-LD) accounts for approximately 80 and 90% of all cases of pulmonary NTM infection in the United States and Japan, respectively, MAC-LD is a chronic and refractory respiratory infection with unclear pathogenesis and unknown risk factors.

A number of epidemiological studies have revealed that patients with MAC-LD are predominantly elderly and postmenopausal women [4, 5]. We previously estimated the MAC infection rate in a healthy population by measuring immunoglobulin (Ig) G antibody titers against the MAC-specific lipid antigen glycopeptidolipid (GPL) [6]. Interestingly, the antibody prevalence in middle-aged to elderly women was significantly higher than that in men or younger women, indicating that middle-aged and elderly women were more likely to be infected with MAC. On the basis of these findings, decreases in sex hormone levels, especially estradiol (E2), after menopause might represent a risk factor for MAC-LD. However, no previous study has verified this hypothesis.

Therefore, we conducted a cross-sectional study to determine whether low serum E2 levels are related to MAC-LD.

## Methods

### Study population

We conducted a cross-sectional study comparing postmenopausal women with MAC-LD with healthy postmenopausal women at a tertiary care center in Japan. Data and samples from outpatients with MAC-LD were obtained between May 2016 and April 2017 from Keio University Hospital, one of the largest referral centers in Tokyo. The hospital treats approximately 400 outpatients annually with MAC-LD in its infectious disease and pulmonary disease clinics. In total, 193 women with MAC-LD provided written informed consent for study participation. The diagnosis of MAC-LD was based on 2007 American Thoracic Society/Infectious Diseases Society of America criteria [7]. Meanwhile, data and samples from healthy controls were obtained through annual health examinations of the faculty and staff of Keio University held at the Mita (Tokyo), Hiyoshi (Kanagawa), and Shonan Fujisawa (Kanagawa) campuses between September 2015 and November 2015. In total, 383 healthy female faculty and staff, who were not healthcare workers, provided written informed consent for study participation.

We obtained a 9-mL blood sample and clinical information through questionnaires and medical records from each study participant. To analyze sex steroid hormones, we included postmenopausal participants aged 65 years or younger because the levels of sex steroid hormones, especially E2, fluctuate considerably with the menstrual cycle and decline with aging. Because 65 years of age is the retirement age for faculty and staff at Keio University, all healthy controls were aged 65 years or younger. Consequently, we excluded patients with MAC-LD who were older than 65 years.

Participants were also excluded if their information was incomplete regarding menopause and the use of sex hormone-based medications such as oral contraceptives and if they used sex-hormone based medications. Additionally, healthy controls with histories of MAC-LD or chest X-ray findings compatible with MAC-LD were excluded. To exclude healthy controls with latent MAC infection without clinical symptoms or chest X-ray abnormality, we measured anti-GPL core IgA antibody titers using a Capilia MAC Ab ELISA kit (TAUNS Laboratories, Inc. Izunokuni, Shizuoka, Japan). GPL is a specific antigen that constitutes the cell wall of MAC; therefore, anti-GPL antibodies are specific for MAC infection [8]. A cutoff value of 0.3 U/mL denoted positivity for anti-GPL core IgA antibodies, [9] and healthy controls with titers exceeding this cutoff were excluded.

To compare the demographic characteristics between the MAC-LD and control groups, we obtained information on age, body mass index (BMI), age at first menstruation, age at menopause, years after menopause, smoking history, and history of tuberculosis, human immunodeficiency virus (HIV) infection, immunosuppression (immunodeficiency or use of immunosuppressive medication), and chronic diseases such as hypertension, diabetes, dyslipidemia, and osteoporosis. Information on age, BMI, history of tuberculosis, HIV, immunosuppression, and history of chronic diseases was obtained from medical and health examination records. Histories of chronic diseases were compiled using medication histories. The ages at first menstruation and menopause, and smoking histories were obtained using a questionnaire. The number of years after menopause was calculated from current age and age at menopause.

To determine if the sex steroid hormone levels were associated specifically with MAC-LD, but not other chronic lung diseases, we conducted an additional study to compare serum sex steroid hormone levels of MAC-LD patients with plasma sex steroid hormone levels of bronchiectasis (BE) patients without NTM infections (non-NTM BE). Pooled blood samples previously obtained for two clinical studies were used. Samples of female MAC-LD patients above 55 years of age, who were diagnosed with MAC-LD based on the 2007 American Thoracic Society/Infectious Diseases Society of America criteria, [7] were selected from pooled serum samples obtained for the “NTM Biomarker Study,” which included serum samples of NTM patients of Keio University Hospital. Meanwhile, samples of female non-NTM BE patients were selected from pooled plasma samples obtained for the “NTM Genome-Wide Analysis Study,” which included plasma samples of NTM patients and BE patients of Keio University Hospital. The following characteristics defined non-NTM BE: 1) clinically diagnosed as BE by respiratory physicians based on CT examination, 2) all previous sputum Mycobacteria culture and smear were negative (at least 2 negative culture results were required), 3) anti-GPL core antibody was not detected.

Because of the small number of non-NTM BE patients, we selected age- (difference within a range of 3 years) and BMI- (difference within a range of 3 kg/m^2^) matched MAC-LD patients and non-NTM BE patients at a ratio of 2:1. Among the matched pairs, a pair of MAC-LD and non-NTM BE patients was excluded if one of the patients was taking oral steroids or sex hormone medication or had a history of ovariectomy or hysterectomy, which might affect sex hormone production.

### Measurement of serum or plasma levels of sex hormones

We measured serum E2 and testosterone levels using a chemiluminescent immunoassay (CLIA) and measured dehydroepiandrosterone sulfate (DHEA-S) levels using a chemiluminescent enzyme immunoassay. If serum E2 levels were under the limit of detection for CLIA (10 pg/mL), a further measurement was performed via liquid chromatography-tandem mass spectrometry.

### Statistical methods

For baseline variables, summary statistics were constructed employing frequencies and proportions for categorical data, and medians and interquartile ranges for continuous variables. We compared patient characteristics using Fisher’s exact test for categorical outcomes and the Mann-Whitney *U* test for continuous variables.

Specifically in the analyses comparing MAC-LD patients with healthy controls, to assess factors independently associated with MAC-LD (i.e., age, BMI, years after menopause, E2, testosterone, and DHEA-S), we additionally performed logistic regression analyses via the simultaneous method. For the analyses, we categorized the serum hormone level of each participant as high or low based on the median hormone level of the study population. Thus, the logistic regression model consisted of three continuous (age, BMI, and years after menopause) and three categorical (low or high E2, testosterone, and DHEA-S) values.

Additionally, to assess the strength of the relationship between MAC-LD and low serum E2 levels, we performed receiver operating characteristic (ROC) curve analysis and calculated the area under the curve (AUC). A *p*-value < 0.05 was considered to be statistically significant. All statistical analyses were performed using SPSS version 25 (IBM, Chicago, IL, USA).

### Age- and BMI-matched subgroup analysis

Additionally, we performed an age- and BMI-matched subgroup analysis of MAC-LD patients and healthy controls, because age and BMI could be strong confounding factors of sex steroid hormones. We selected age- (exact match) and BMI- (difference within a maximum range of 2 kg/m^2^) matched MAC-LD patients and healthy controls at a ratio of 1:1. Using this matched subgroup, we performed ROC curve analysis.

## Results

### Study population

From the original dataset, 42 patients with MAC-LD and 101 healthy controls fulfilled the inclusion criteria. Three healthy controls using hormone medications were excluded. In addition, seven healthy controls with histories of MAC-LD or anti-GPL core IgA antibody positivity were excluded. Thus, the analysis ultimately included 42 patients with MAC-LD and 91 healthy controls.

The patients with MAC-LD were significantly older than the healthy controls (61 years vs. 56 years, *p* < 0.001), whereas BMI was significantly lower in the MAC-LD group (19.14 kg/m^2^ vs. 22.18 kg/m^2^, *p* < 0.001). There were no significant differences regarding the age at first menstruation, menopausal age, smoking history, or history of tuberculosis and chronic diseases between the two groups (Table 1). There were no patients with HIV or immunosuppression except for one patient in the MAC-LD group taking methotrexate for rheumatoid arthritis.

**Table 1:** Demographic characteristics of the study subjects

	MAC-LD (*n* = 42)	Control (*n* = 91)	*p*-value^†^
Age, years, median (IQR)	61 (59–64)	56 (54–59)	< 0.001
BMI, median (IQR)	19.14 (17.85–20.58)	22.18 (19.68–24.53)	< 0.001
Age at menopause, years, median (IQR)	50.5 (50–53)	51 (49–53)	0.798
Age at first menstruation, years, median (IQR)	12.5 (12–13.75)	12 (12–13)	0.383
Years after menopause, years, median (IQR)	11.5 (8–12.75)	5 (2–8.25)	< 0.001
History of tuberculosis, n (%)	1 (2.4)	4 (4.4)	1.000
Chronic diseases, n (%)	10 (23.8)	32 (35.2)	0.231
Hypertension, n (%)*	3 (7.1)	16 (17.5)	0.181
Diabetes mellitus, n (%)*	1 (2.4)	3 (3.3)	1.000
Dyslipidemia, n (%)*	2 (4.8)	17 (18.7)	0.035
Osteoporosis, n (%)*	5 (11.9)	3 (3.3)	0.108
History of smoking, n (%)	8 (19.0)	17 (18.7)	1.000

Abbreviations: *MAC-LD Mycobacterium avium* complex lung disease, *BMI* body mass index, *IQR* interquartile range

*Chronic diseases that required medication

^†^Mann-Whitney *U* test for continuous variables and Fisher’s exact test for categorical variables

### Serum levels of sex hormones

**Fig. 1:**
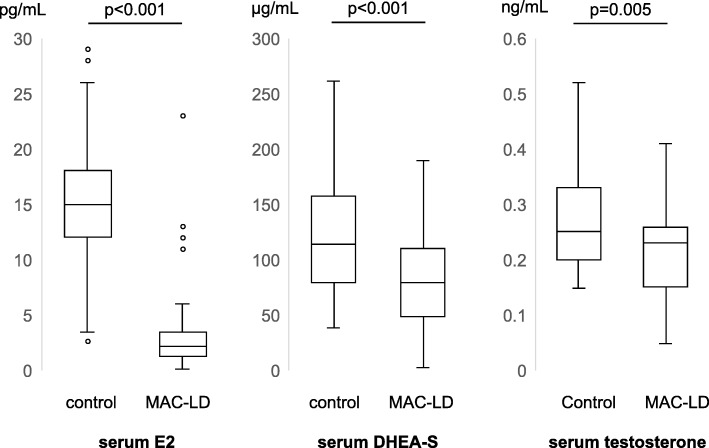
Box plots* of serum sex hormones of MAC-LD patients and healthy controls. Abbreviation: MAC-LD = *Mycobacterium avium* complex lung disease. *Mann-Whitney *U* test

Serum E2 (2.20 pg/mL vs. 15.0 pg/mL, *p* < 0.001), testosterone (0.23 ng/L vs. 0.25 ng/L, *p* = 0.005), and DHEA-S (82.5 μg/dL vs. 114.0 μg/dL, *p* < 0.001) levels were significantly lower in the MAC-LD group than in the control group (Fig. 1).

### Low serum E2 levels were independently related to MAC-LD

**Fig. 2:**
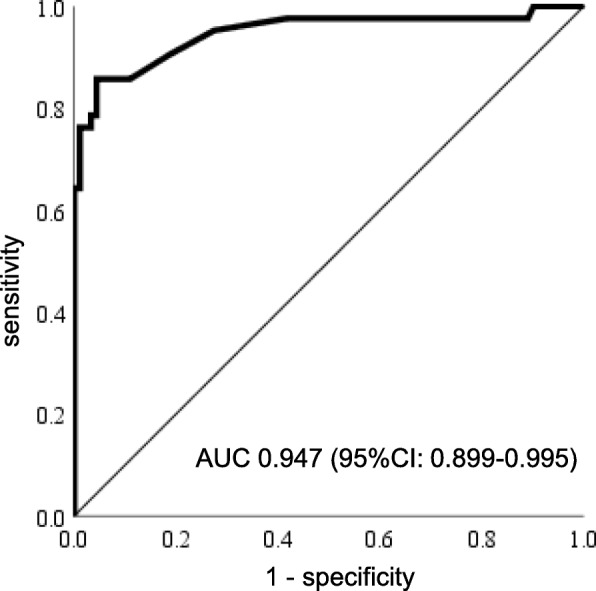
ROC curve of estradiol serum levels in samples from MAC-LD patients and healthy controls. Abbreviations: ROC = receiver operating characteristics, MAC-LD = *Mycobacterium avium* complex lung disease, AUC = area under the curve, CI = confidence interval

According to the logistic regression analysis, age and low serum E2 levels were independently related to MAC-LD (Table 2). As expected, the number of years after menopause was strongly correlated with age (Spearman’s rank correlation coefficient; r = 0.727, *p* < 0.001); therefore, the number of years after menopause was not included in the logistic regression analysis. To determine the strength of the correlation between serum E2 levels and MAC-LD, ROC curve analysis was performed (Fig. 2). The AUC was 0.947 (95% confidence interval = 0.899–0.995).

**Table 2:** Multivariate analysis of the relationships between MAC-LD and age, BMI, and sex hormones

	OR	95% CI	adjusted OR	95% CI
Age	1.42	1.25–1.62	1.50	1.23–1.81
BMI	0.77	0.67–0.89	0.87	0.72–1.05
Low DHEA-S	2.05	0.98–4.32	1.17	0.35–3.95
Low testosterone	1.78	0.85–3.72	0.94	0.28–3.21
Low E2	52.80	11.87–234.95	34.62	6.02–199.14

Abbreviations: *MAC-LD Mycobacterium avium* complex lung disease, *OR* odds ratio, *CI* confidence interval, *BMI* body mass index, *DHEA-S* dehydroepiandrosterone sulfate, *E2* estradiol

### Low serum E2 levels were related to MAC-LD even in age- and BMI-matched subgroups

**Fig. 3:**
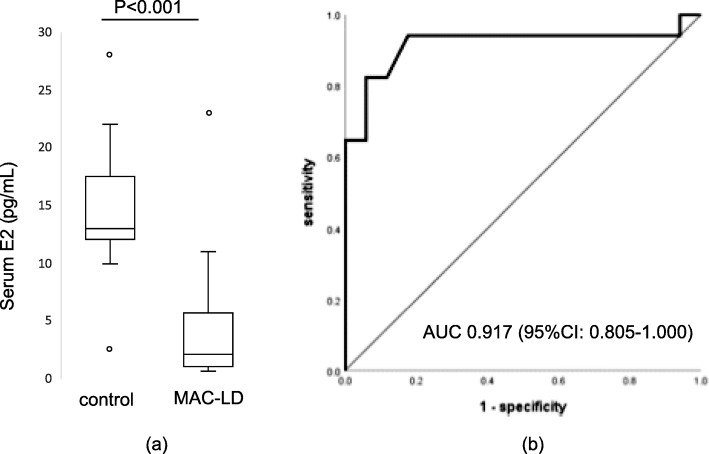
Results of age- and BMI-matched subgroup analysis. **a** Box plot* of serum estradiol of age- and BMI-matched MAC-LD patients and healthy controls. **b** ROC curve of estradiol serum levels in samples from age- and BMI-matched MAC-LD patients and healthy controls. Abbreviations: BMI = body mass index, MAC-LD = *Mycobacterium avium* complex lung disease, ROC = receiver operating characteristics, AUC = area under the curve, CI = confidence interval. *Mann-Whitney *U* test

Among 42 patients with MAC-LD and 91 healthy controls, 17 patients with MAC-LD and 17 healthy controls were matched (Table 3). Even after age and BMI were matched, serum E2 (2.05 pg/mL vs. 13.0 pg/mL, *p* < 0.001) was lower in MAC-LD patients than in healthy controls (Fig. 3a). ROC curve analysis showed that the AUC of serum E2 was 0.917 (95% confidence interval = 0.805–1.000) (Fig. 3b).

**Table 3:** Demographic characteristics of age- and BMI-matched case controls

	MAC-LD (*n* = 17)	Control (n = 17)	*p-*value^†^
Age, years, median (IQR)	59 (58–60.5)	59 (58–60.5)	1.000
BMI, median (IQR)	19.35 (17.81–20.80)	20.36 (19.00–21.08)	0.394
Age at menopause, years, median (IQR)	50 (50–52)	52 (49–53)	0.737
Age at first menstruation, years, median (IQR)	12 (11–13)	12 (11–13)	1.000
Years after menopause, years, median (IQR)	8 (5–12)	7 (5–10.5)	0.737
History of tuberculosis, n (%)	0 (0.0)	1 (5.9)	1.000
Chronic diseases, n (%)	4 (23.5)	7 (41.2)	0.465
Hypertension, n (%)*	1 (5.9)	3 (17.6)	0.601
Diabetes mellitus, n (%)*	1 (5.9)	1 (5.9)	1.000
Dyslipidemia, n (%)*	2 (11.8)	4 (23.5)	0.656
Osteoporosis, n (%)*	1 (5.9)	1 (5.9)	1.000
History of smoking, n (%)	4 (23.5)	3 (17.6)	1.000

Abbreviations: *MAC-LD Mycobacterium avium* complex pulmonary disease, *BMI* body mass index, *IQR* interquartile range

*Chronic diseases that required medication

^†^Mann-Whitney *U* test for continuous variables and Fisher’s exact test for categorical variables

### Median E2 level of MAC-LD patients was lower than that of non-NTM BE patients

We used 88 pooled samples of MAC-LD patients obtained from Sep 2015 to Jun 2018 and 18 pooled samples of non-NTM BE patients obtained from Nov 2017 to Oct 2018. Samples from 20 patients with MAC-LD and samples from 11 non-NTM BE patients were compared. Consistent with matching, we found that the median age (74 years vs 76 years, *p* = 0.699) and BMI (18.98 kg/m^2^ vs 19.27 kg/m^2^, *p* = 0.984) of MAC-LD patients were equivalent to those of non-NTM BE patients. Median serum E2 levels in MAC-LD patients were significantly lower than median plasma E2 levels in non-NTM BE patients (1.79 pg/mL vs. 11.0 pg/mL, *p* < 0.001), while DHEA-S and testosterone were not significantly different between the two groups (Table 4).

**Table 4:** Demographic characteristics and sex hormone levels compared between patients with MAC-LD and non-NTM BE

	MAC-LD (*n* = 20)	Non-NTM BE (*n* = 11)	*p-*value^†^
Age, years, median (IQR)	74 (64.25–77.75)	76 (65–78)	0.699
BMI, median (IQR)	18.98 (17.65–20.97)	19.27 (17.29–21.62)	0.984
E2, pg/mL, median (IQR)	1.79 (1.20–2.47)	11.0 (2.77–13.0)	< 0.001
DHEA-S, μg/mL, median (IQR)	51.5 (39–97)	85 (53–99)	0.145
Testosterone, ng/mL, median (IQR)	0.185 (0.152–0.255)	0.220 (0.180–0.260)	0.359

Abbreviations: *MAC-LD Mycobacterium avium* complex lung disease, *non-NTM BE* bronchiectasis without non-tuberculous mycobacteria infection, *BMI* body mass index, *IQR* interquartile range

*Chronic diseases that required medication

^†^Mann-Whitney *U* test for continuous variables and Fisher’s exact test for categorical variables

## Discussion

The incidence of pulmonary NTM infection has increased worldwide, and most cases are linked to MAC-LD. However, no effective treatment or preventive modality has been developed for MAC-LD because its pathogenesis is unclear. As most patients with MAC-LD are postmenopausal women, we speculated that low serum E2 levels represent a potential risk factor for MAC-LD. Our cross-sectional study revealed a link between low serum E2 levels and MAC-LD in postmenopausal women aged 65 years and younger. The age- and BMI-matched analyses also showed the same trends. Additionally, low serum E2 levels were seen in MAC but not in non-NTM BE, in women approximately 75 years-old, suggesting that low serum E2 was related specifically to MAC-LD.

Although we did not study causal relationships between low serum levels and MAC-LD, it is possible that E2 is protective against MAC infection. Tsuyuguchi et al. reported that ovariectomized mice had a higher MAC burden in the lungs 3–6 weeks after infection than sham-operated mice and E2-treated ovariectomized mice [10]. Calippe et al. found that long-term E2 administration to ovariectomized mice increased interleukin-1 beta secretion and inducible nitric oxide synthase expression which enhanced the killing of intracellular mycobacteria by resident peritoneal macrophages after lipopolysaccharide stimulation [11]. These reports indicate that E2 plays a protective role against MAC infection, in line with our findings. However, to demonstrate the protective role of E2 against MAC infection, further experimental studies are essential. For instance, the change in phagocytic and bactericidal abilities with or without E2 in human macrophages could be explored. Additionally, assessing the effects of changes in body weight, lung weight, and lung pathology using a MAC-infected aromatase-knockout mouse (which cannot produce E2 in either the ovary or peripherally) might reinforce our conclusion.

Danley et al. found that women with MAC-LD had lower serum DHEA-S levels but not lower serum estrogen levels than control subjects without lung disease [12]. However, our results identified an association between lower serum E2 levels and MAC-LD in postmenopausal women. Currently, we are unable to explain these divergent findings. To verify which sex hormone might affect host susceptibility to MAC-LD, additional research with larger sample size and age stratification is necessary. However, according to reports that DHEA is transformed to E2 by aromatase in macrophages, [13, 14] one could the hypothesize that both DHEA and estrogen have protective effects against MAC infection.

The ROC curve analysis suggested a strong association between MAC-LD and E2. Furthermore, the high AUC makes E2 a potential candidate in developing diagnostic tools. Although it is difficult to diagnose MAC-LD without microbiological findings and imaging, low E2 levels might indicate pretest probabilities of MAC-LD.

The findings of this study imply that controlling sex hormones could be a new approach for preventing or treating MAC-LD. Although additional research is required to uncover mechanisms and demonstrate causal relationships between low E2 levels and MAC-LD, measuring E2 and prescribing hormone replacement therapy for postmenopausal women with low E2 levels could be ancillary prevention and treatment option for MAC-LD patients.

The present study had several limitations. First, because the study sample was restricted to women no older than 65 years in the study comparison with healthy controls, the external validity of the study is limited. We needed healthy controls older than 65 years to solve this problem, however, it was slightly difficult to recruit a large number of “healthy” elderly women because the opportunity to access medical facilities of such a population were limited. We, therefore, included the additional study comparing MAC-LD with non-NTM BE, partially because this study population was approximately 75 years-old and demonstrated the same trends. Second, circadian rhythms might affect sex steroid hormone levels, especially that of testosterone, which is believed to be high in the morning and low in the evening. Third, it was uncertain whether low E2 levels were associated only with MAC-LD or also linked to chronic lung infections. To answer this question, further research comparing patients with MAC-LD to those with other chronic lung infections such as tuberculosis, aspergillosis or *Mycobacterium abscessus* lung infections and healthy controls is necessary. Fourth, hormones measured in the non-NTM BE group were not from serum but plasma. We do not believe this affected our result because the difference between serum and plasma E2 levels is little according to previous studies measuring serum and plasma E2 at the same time [15, 16]. Finally, it is unclear whether the decline in serum E2 levels precedes MAC-LD or whether MAC-LD reduces E2 concentrations. Future well-powered prospective and longitudinal studies may resolve these issues.

## Conclusion

Low serum E2 levels were independently related to MAC-LD in middle-aged and presenile postmenopausal women.

## Data Availability

The datasets used and/or analyzed during the current study are available from the corresponding author on reasonable request.

## Abbreviations

AUC: area under the curve
BE: bronchiectasis
BMI: body mass index
CLIA: chemiluminescent immunoassay
DHEA-S: dehydroepiandrosterone sulfate
E2: estradiol
GPL: glycopeptidolipid
HIV: human immunodeficiency virus infection
Ig: immunoglobulin
MAC: *Mycobacterium avium* complex
MAC-LD: *Mycobacterium avium* complex lung disease
NTM: nontuberculous mycobacteria
ROC: receiver operating characteristic
