# Correction to: Low serum estradiol levels are related to *Mycobacterium avium* complex lung disease: a cross-sectional study

**DOI:** 10.1186/s12879-020-4868-4

**Published:** 2020-02-12

**Authors:** Yoshifumi Uwamino, Tomoyasu Nishimura, Yasunori Sato, Eiko Tamizu, Takanori Asakura, Shunsuke Uno, Masaaki Mori, Hiroshi Fujiwara, Makoto Ishii, Hiroshi Kawabe, Mitsuru Murata, Naoki Hasegawa

**Affiliations:** 1 Department of Laboratory Medicine, Keio University School of Medicine, 35 Shinanomachi, Shinjuku-ku, Tokyo, Japan; 2 Department of Infectious Diseases, Keio University School of Medicine, 35 Shinanomachi, Shinjuku-ku, Tokyo, Japan; 3 Keio University Health Center, 35 Shinanomachi, Shinjuku-ku, Tokyo, 160-8582 Japan; 4 Department of Preventive Medicine and Public Health, Keio University School of Medicine, 35 Shinoanomachi, Shinjuku-ku, Tokyo, Japan; 5 Division of Pulmonary Medicine, Department of Medicine, Keio University School of Medicine, 35 Shinanomachi, Shinjuku-ku, Tokyo, Japan; 6 Department of Internal Medicine, Keio University School of Medicine, 35 Shinanomachi, Shinjuku-ku, Tokyo, Japan

**Correction to: BMC Infect Dis**

**https://doi.org/10.1186/s12879-019-4668-x**

After publication of the original article [1], we were notified that units of testosterone in main text and abstract and units of DHEA-S in Fig. 1 and Table 4 are incorrect.

1. In the Abstract:
  ‘testosterone (0.230 **ng/L** vs. 0.250 **ng/L**, p = 0.005)’ should be replaced with ‘testosterone (0.230 **ng/mL** vs. 0.250 **ng/mL**, p = 0.005)’
2. In the section ‘Serum levels of sex hormones’:
  ‘testosterone (0.23 **ng/L** vs. 0.25 **ng/L**, p = 0.005)’ should be replaced with ‘testosterone (0.23 **ng/mL** vs. 0.25 **ng/mL**, p = 0.005)’
3. The unit of serum DHEA-S, in Fig. 1 (3rd column):
  **‘**μg**/mL**’ should be replaced with ‘**μg/dL’**
4. unit of DHEA-S in Table 4 (4th row):
  ‘**μg/mL**’ should be replaced with ‘**μg/dL’**
